# Supplementary material for: Acceptability of improved cook stoves-a scoping review of the literature
Source: PLOS Glob Public Health. 2025 Jan 7;5(1):e0004042. doi: 10.1371/journal.pgph.0004042 (PMC11706475; doi:10.1371/journal.pgph.0004042)
Supplement: S2 File — (DOCX) [file pgph.0004042.s002.docx]

Acceptability of improved cook stoves-a scoping review of the literature

Sophie Suh Young Kang^1^, Aaryan Dahal^1^, Salum Mshamu^2,3^, Jacqueline Deen^4^, Thomas Chevalier Bøjstrup^5^, Jakob Knudsen^5^, Christopher Pell^6,7,8^, Lorenz von Seidlein^1,3^, Bipin Adhikari^1,3*^

^1^Mahidol Oxford Tropical Medicine Research Unit, Faculty of Tropical Medicine, Mahidol University, Bangkok, Thailand

^2^CSK Research Solutions, Mtwara, Tanzania

^3^Centre for Tropical Medicine and Global Health, Nuffield Department of Medicine, University of Oxford, United Kingdom

^4^University of Philippines, Manila, Philippines

^5^Royal Danish Academy – Architecture, Design, Conservation, Copenhagen, Denmark

^6^Amsterdam University Medical Centres, University of Amsterdam, Department of Global Health, Amsterdam, the Netherlands

^7^Amsterdam Institute for Global Health and Development, Amsterdam, The Netherlands

^8^Amsterdam Public Health Research Institute, Amsterdam, the Netherlands

*Corresponding author: Bipin@tropmedres.ac

Literature search strategy (last updated on April 2024)

**Medline**

1. Cook* and ("clean cook*" or Electric* or "Induction stove" or Biogas or Biodigester or Biofuel or "Liquid petroleum gas" or "Liquefied petroleum gas" or LPG or "Improved cooking stove" or ICS or Ethanol or "Modern fuel" or "Clean fuel" or Solar or Photovoltaic or Briquette or Pellet or "Natural gas" or LNG or "Gasifier stove" or Subsidy)
2. Accept* or Adopt* or Use* or Usage or Uptake or Choose* or Choice or Switch or Replace* or Substitut*
3. focus groups[MESH:NOEXP] OR "interviews as topic"[MESH:NOEXP] OR narration[MESH:NOEXP] OR qualitative research[MESH:NOEXP] OR ethnograph*[TIAB] OR "field work"[TIAB] OR fieldwork[TIAB] OR focus group*[TIAB] OR key informant*[TIAB] OR qualitative[TIAB] OR (face-to-face[TIAB] OR guided[TIAB] OR in-depth[TIAB] OR indepth[TIAB] OR semi-structured[TIAB] OR structured[TIAB] OR unstructured[TIAB]) AND (discussion*[TIAB] OR interview*[TIAB] OR questionnaire*[TIAB] OR informal[TIAB] OR “key informant”[TIAB] OR "personal narratives as topic"[Mesh]))
4. #1 AND #2 AND #3

**Scopus**

1. TITLE-ABS-KEY (Cook* and ("clean cook*" or Electric* or "Induction stove" or Biogas or Biodigester or Biofuel or "Liquid petroleum gas" or "Liquefied petroleum gas" or LPG or "Improved cooking stove" or ICS or Ethanol or "Modern fuel" or "Clean fuel" or Solar or Photovoltaic or Briquette or Pellet or "Natural gas" or LNG or "Gasifier stove" or Subsidy))
2. TITLE-ABS-KEY (Accept* or Adopt* or Use* or Usage or Uptake or Choose* or Choice or Switch or Replace* or Substitut*)
3. TITLE-ABS-KEY (qualitative* OR ethnol* OR ethnog* OR ethnonurs* OR emic OR etic OR leininger OR noblit OR "field note*" OR "field record*" OR fieldnote* OR "field stud*" or "participant observ*" OR "participant observation*" OR hermaneutic* OR phenomenolog* OR "lived experience*" OR heidegger* OR husserl* OR "merleau-pont*" OR colaizzi OR giorgi OR ricoeur OR spiegelberg OR "van kaam" OR "van manen" OR "grounded theory" OR "constant compar*" OR "theoretical sampl*" OR (glaser AND strauss) OR "content analy*" OR "thematic analy*" OR narrative* OR "unstructured categor*" OR "structured categor*" OR "unstructured interview*" OR "semi-structured interview*" OR "maximum variation*" OR snowball OR audio* OR tape* OR video* OR metasynthes* OR "meta-synthes*" OR metasummar* OR "meta-summar*" OR metastud* OR "meta-stud*" OR "meta-ethnograph*" OR metaethnog* OR "meta-narrative*" OR metanarrat* OR " meta-interpretation*" OR metainterpret* OR "qualitative meta-analy*" OR "qualitative metaanaly*" OR "qualitative metanaly*" OR "purposive sampl*" OR "action research" OR "focus group*" or photovoice or "photo voice" or  "mixed method*")
4. #1 AND #2 AND #3

**Web of Science**

1. TOPIC (Cook* and ("clean cook*" or Electric* or "Induction stove" or Biogas or Biodigester or Biofuel or "Liquid petroleum gas" or "Liquefied petroleum gas" or LPG or "Improved cooking stove" or ICS or Ethanol or "Modern fuel" or "Clean fuel" or Solar or Photovoltaic or Briquette or Pellet or "Natural gas" or LNG or "Gasifier stove" or Subsidy))
2. TOPIC (Accept* or Adopt* or Use* or Usage or Uptake or Choose* or Choice or Switch or Replace* or Substitut*)
3. TOPIC (qualitative* OR ethnol* OR ethnog* OR ethnonurs* OR emic OR etic OR leininger OR noblit OR "field note*" OR "field record*" OR fieldnote* OR "field stud*" or "participant observ*" OR "participant observation*" OR hermaneutic* OR phenomenolog* OR "lived experience*" OR heidegger* OR husserl* OR "merleau-pont*" OR colaizzi OR giorgi OR ricoeur OR spiegelberg OR "van kaam" OR "van manen" OR "grounded theory" OR "constant compar*" OR "theoretical sampl*" OR (glaser AND strauss) OR "content analy*" OR "thematic analy*" OR narrative* OR "unstructured categor*" OR "structured categor*" OR "unstructured interview*" OR "semi-structured interview*" OR "maximum variation*" OR snowball OR audio* OR tape* OR video* OR metasynthes* OR "meta-synthes*" OR metasummar* OR "meta-summar*" OR metastud* OR "meta-stud*" OR "meta-ethnograph*" OR metaethnog* OR "meta-narrative*" OR metanarrat* OR " meta-interpretation*" OR metainterpret* OR "qualitative meta-analy*" OR "qualitative metaanaly*" OR "qualitative metanaly*" OR "purposive sampl*" OR "action research" OR "focus group*" or photovoice or "photo voice" or  "mixed method*")
4. #1 AND #2 AND #3
