# Supplementary material for: Acceptability of improved cook stoves-a scoping review of the literature
Source: PLOS Glob Public Health. 2025 Jan 7;5(1):e0004042. doi: 10.1371/journal.pgph.0004042 (PMC11706475; doi:10.1371/journal.pgph.0004042)
Supplement: Attachment — Submitted filename: RESPONSE TO REVIEWERS COMMENTS.docx [file pgph.0004042.s005.docx]

5^th^ November 2024

Dear Prof. Naveen Puttaswamy, Prof. Sankar Sambandam, and Prof. Rachit Sharma

Thank you very much for your suggestion and opportunity to revise our work. We have utilized your suggestions to improve our manuscript. Please see below our responses to your specific suggestions with corresponding changes in the manuscript.

We look forward to your kind consideration.

On behalf of all the authors,

Bipin Adhikari

PGPH-D-24-01963
Acceptability of improved cook stoves--a scoping review of the literature
PLOS Global Public Health

Dear Dr. Adhikari,

Thank you for submitting your manuscript to PLOS Global Public Health. After careful consideration, we feel that it has merit but does not fully meet PLOS Global Public Health’s publication criteria as it currently stands. Therefore, we invite you to submit a revised version of the manuscript that addresses the points raised during the review process.


Please submit your revised manuscript by Dec 07 2024 11:59PM. If you will need more time than this to complete your revisions, please reply to this message or contact the journal office at [globalpubhealth@plos.org](mailto:globalpubhealth@plos.org). When you're ready to submit your revision, log on to [https://www.editorialmanager.com/pgph/](https://url.uk.m.mimecastprotect.com/s/SQFnCOElQFpZORkiEfYFG__hO?domain=editorialmanager.com) and select the 'Submissions Needing Revision' folder to locate your manuscript file.

Please include the following items when submitting your revised manuscript:

- A rebuttal letter that responds to each point raised by the editor and reviewer(s). You should upload this letter as a separate file labeled 'Response to Reviewers'.
- A marked-up copy of your manuscript that highlights changes made to the original version. You should upload this as a separate file labeled 'Revised Manuscript with Track Changes'.
- An unmarked version of your revised paper without tracked changes. You should upload this as a separate file labeled 'Manuscript'.

Guidelines for resubmitting your figure files are available below the reviewer comments at the end of this letter.

We look forward to receiving your revised manuscript.

Kind regards,

Naveen Puttaswamy, Ph.D
Academic Editor
PLOS Global Public Health

Journal Requirements:

Additional Editor Comments (if provided):


[Note: HTML markup is below. Please do not edit.]

Reviewers' comments:

Reviewer's Responses to Questions

**Comments to the Author**

1. Does this manuscript meet PLOS Global Public Health’s [publication criteria](https://url.uk.m.mimecastprotect.com/s/j3SuCPMmRCKN6Gnh0hRFxcqDe?domain=track.editorialmanager.com)? Is the manuscript technically sound, and do the data support the conclusions? The manuscript must describe methodologically and ethically rigorous research with conclusions that are appropriately drawn based on the data presented.

Reviewer #1: Yes

Reviewer #2: Partly

2. Has the statistical analysis been performed appropriately and rigorously?

Reviewer #1: I don't know

Reviewer #2: N/A

3. Have the authors made all data underlying the findings in their manuscript fully available (please refer to the Data Availability Statement at the start of the manuscript PDF file)?

The [PLOS Data policy](https://url.uk.m.mimecastprotect.com/s/XnoLCQNJVfklRALTMivFGZogF?domain=track.editorialmanager.com) requires authors to make all data underlying the findings described in their manuscript fully available without restriction, with rare exception. The data should be provided as part of the manuscript or its supporting information, or deposited to a public repository. For example, in addition to summary statistics, the data points behind means, medians and variance measures should be available. If there are restrictions on publicly sharing data—e.g. participant privacy or use of data from a third party—those must be specified.

Reviewer #1: Yes

Reviewer #2: Yes

4. Is the manuscript presented in an intelligible fashion and written in standard English?

PLOS Global Public Health does not copyedit accepted manuscripts, so the language in submitted articles must be clear, correct, and unambiguous. Any typographical or grammatical errors should be corrected at revision, so please note any specific errors here.

Reviewer #1: Yes

Reviewer #2: Yes

5. Review Comments to the Author

Please use the space provided to explain your answers to the questions above. You may also include additional comments for the author, including concerns about dual publication, research ethics, or publication ethics. (Please upload your review as an attachment if it exceeds 20,000 characters)

**Reviewer #1**

**The title is concise and research question is clearly identified. The main elements of the topic are covered, however few topics need to be elaborated as presented in the comments. The number of studies identified and selected for the scoping review is discussed and clearly detailed in the flow chart (figure 1). In the figure 1, the reason for records excluded (n=1671) may be briefly mentioned as specified in other boxes. The authors shall identify gaps and future areas of research especially in the ICS context.**

**AUTHORS:** Thank you for the suggestion. We have revised the figure 1 to add the reasons for screening out articles by title and abstract.

**Interventions addressing indoor air pollution
1. Multi-pronged interventions, such as improved ventilation, separate cooking
spaces, and environmentally friendly fuels seek to minimise HAP [4].

Comment: Do the reference cited only presented the three types of interventions or if the authors have not discussed about the ICS?**

**AUTHORS:** Thank you for the question. Since this was still at the introduction section, we were only attempting to introduce how multi-pronged interventions are designed to reduce the household air pollution through for example, improve ventilation, separate cooking spaces, and environmentally friendly fuels for cooking.

**What is improved cooking stove?
2. biomass stoves produced from local low-cost materials (e.g. cement stove);

Comments: Elsewhere in the world, especially in India, stoves are also constructed with earthen material. The authors shall clarify by including this.**

**AUTHORS:** Thank you for the suggestion. We agree and have added in the revised paragraph and reads as follows:

Improved cook-stoves (ICSs) bear a wide-spectrum of advances including their sources of fuel. Broadly, stoves can be categorized into 1. three-stone stoves that uses biomass fuels (for e.g. charcoal, crop residue, animal dung and wood); 2. biomass stoves produced from local low-cost materials (e.g. earthen material, and cement); 3. improved biomass stoves using newer technologies for cleanliness, better combustion and safety; 4. advanced biomass stoves that use a fan to force emission back into flame for better combustion (e.g. forced air stoves, and gasifier stoves); and 5. clean fuel stoves using clean and efficient fuels such as LPG, biogas, cooking gas, electricity and solar energy [5-7].
**3. improved biomass stoves using newer technologies for cleanliness and safety;

Comment: Is new technologies are for cleanliness and safety? It shall be better combustion as well. Please clarify**

**AUTHORS:** Thank you for the suggestion. We agree, we have added in our revised paragraph to clarify that, and reads as follows:

Improved cook-stoves (ICSs) bear a wide-spectrum of advances including their sources of fuel. Broadly, stoves can be categorized into 1. three-stone stoves that uses biomass fuels (for e.g. charcoal, crop residue, animal dung and wood); 2. biomass stoves produced from local low-cost materials (e.g. earthen material, and cement); 3. improved biomass stoves using newer technologies for cleanliness, better combustion and safety; 4. advanced biomass stoves that use a fan to force emission back into flame for better combustion (e.g. forced air stoves, and gasifier stoves); and 5. clean fuel stoves using clean and efficient fuels such as LPG, biogas, cooking gas, electricity and solar energy [5-7].

**4. Fuel sources can be categorized into biomass (firewood, crop residue, animal dung, and charcoal), transitional fuel (kerosene) and more modern, cleaner fuels (electricity, solar energy and liquefied petroleum gas) [6].

Comment: Do the author agree that there are biomass pellet/processed type of fuel? I see the literature review have not captured this. Please clarify.**

**AUTHORS:** Thank you for the suggestion. We agree that biomass pellets are produced to increase the energy and mass density of residue feedstocks, wood, wood-saw dust, grass, hay, rice husk which makes them easy to store and transport. We have added in our revised statement and reads as follows:

Fuel sources can be categorized into biomass (firewood, crop residue, animal dung, charcoal and various types of pellets), transitional fuel (kerosene) and more modern, cleaner fuels (electricity, solar energy and liquefied petroleum gas) [8].

**5. Comment: In the ICS section, the authors have not discussed about the types of improved cook stoves. The article DOI: 10.1007/s10393-014-0976-1 and 10.1016/j.aogh.2015.08.009 presents about testing various types of commercial stoves. Similarly, there should be other types of stoves elsewhere (eg. rocket stoves). Additional literatures shall be looked into it. Please clarify.**

**AUTHORS:** Thank you for the suggestion. We acknowledge your suggested article, and we have revised the statement to clarify the statement and added the reference you suggested including other one about rocket stoves. Since our main goal was to explore the acceptability of improved cook stove—operationally defined for the purpose of the review, we streamlined our background section towards the acceptability. Based on your suggestion, we have added/revised in the introduction and discussion section as well. The changes are documented as follows:

Introduction section, under the heading: What is improved cooking stove?

Improved cook-stoves (ICSs) bear a wide-spectrum of advances including their sources of fuel. Broadly, stoves can be categorized into 1. three-stone stoves that uses biomass fuels (for e.g. charcoal, crop residue, animal dung and wood); 2. biomass stoves produced from local low-cost materials (e.g. earthen material, and cement); 3. improved biomass stoves using newer technologies for cleanliness, better combustion and safety; 4. advanced biomass stoves that use a fan to force emission back into flame for better combustion (e.g. forced air stoves, and gasifier stoves); and 5. clean fuel stoves using clean and efficient fuels such as LPG, biogas, cooking gas, electricity and solar energy [5-8].

Discussion section, third paragraph reads as follows:

Cleaner fuels are increasingly used but their adoption depends on the functionality of the stove, cooking capacity, ease of use based on their designs, the required size for pots and pans, family or group size to be catered for and traditional food items that are thought to require firewood [8, 29, 35, 39, 102].

**What are the current literature findings around ICS acceptability?
6. Two dominant theories explain the adoption of energy options namely ‘energy ladder’ and ‘energy stacking’ [8-11]**

**Comment: Can theories be reworded? Later in the discussion the authors have used “two broader approaches were described a) the energy ladder that proposes use of energy to be dependent on economic caliber and b) stacking fuel for security purposes [8]. Theory might mean a different aspect. Can this be reworded?**

**AUTHORS:** Thank you for the suggestion. We agree with you. We replaced the word theory by ‘concept’ throughout the manuscript so that it avoids the misinterpretation.

**Fuel
7. Comment: In the last paragraph, some narrations on fuel use in other countries shall be included.**

**AUTHORS:** Thank you for the suggestion. Based on your suggestion, we have revised the last paragraph within the section. We have added countries from Indian sub-continent including countries from outside the Indian sub-continent that included China, Indonesia and Cambodia and reads as follows:

The urban/rural divide for cooking fuel did not apply invariably. For example in some rural areas of Peru, the predominant fuel for cooking was LPG (66%) followed by wood (29%) and the remaining 5% were coal, natural gas and electricity [105]. By contrast, some urban areas in Rwanda used biomass as a predominant (80.3%) source of fuel [66]. A comparative three country-study found that in rural households, women were more likely to burn locally available biomass, such as cow, sheep, and alpaca dung and firewood in Peru; firewood, and crop residue in Kenya; and firewood, dung, crop residue and coal in Nepal [90]. Studies from India [69, 71, 73, 79, 80, 82, 109], Pakistan [78, 97], Bangladesh [72, 88, 89], and Nepal [91, 92], reported predominantly use of firewood or biomass fuels, although the urban-rural division in terms of fuel choice was increasing over the years, including the use of multiple fuel types [67, 86, 87, 95]. The stacking of fuel types including transitioning to cleaner fuel was also reported from China [75], Indonesia [5, 94] and Cambodia [5]. There was a general trend to adopt cleaner fuels globally. For instance, in Guatemala, 50.1% of urban households and 93.4% of rural households use wood for cooking, but households were gradually adopting LPG as a main source of fuel [107].

**8. Comment: Preferences of fuel use is not discussed in this section. People generally use biomass stove for animal fodder preparation, water boiling for bathing and large meal preparation. The author shall narrate on this.**

**AUTHORS:** Thank you for the suggestion. We attempted to present the type of fuel use for cooking, rural-urban divide including the general trend in the section ‘type of fuel’. In the subsequent theme, ‘number of burner and size’ we present the findings related to demands (number of family size), food types (social event, animal food preparation), including other utilities (water boiling, and heating the home). We have revised the section to include your suggestion, and reads as follows:

Traditional stoves (e.g. three stone stoves and biomass fuels) were also used to warm the house during winter months, heating water for bathing, and drinking [46, 62, 92]; including to deter insects, and pests by the smoke produced [77].

**Kitchen architecture
9. Comment: There are different types of kitchen configuration. For example, outdoor cooking, indoor with separate kitchen, indoor with attached kitchen, indoor with no separate kitchen area etc. Several literatures have been published on this. The authors shall narrate on the implication of different types of kitchen on ICS use.**

**AUTHORS:** Thank you for the suggestion. We have revised the section ‘Kitchen architecture’ adding more details from the included studies that showed relationship between use of ICS and cleaner fuels with kitchen’s configuration. We agree with you that acceptability and use of ICS may be affected by indoor details of the kitchen; the studies included by search strategy in this review echoed how outdoor kitchen were more common in rural areas, and indoor kitchen were more common in urban areas which meant the use of solid fuel was difficult within the indoor kitchens. Even when residents live in a single room apartment with no separate kitchen, they are bound to use cleaner fuels, e.g. students in urban areas. Similarly, in big and luxurious house with attached kitchen in urban areas, the residents are also likely to use the cleaner fuels (e.g. LPG or electricity), or more advanced ICS but at the same time, if they have outdoor kitchen and spaces, they may simultaneously use solid fuels (e.g. coals for barbecue). We agree that kitchen configuration (type, space, and design) thus can affect the use of ICS in a spectrum. The revised paragraph reads as follows:

The available space made a major impact on type of stoves used [38, 55, 68, 97]. In urban houses without outdoor kitchens for burning firewood, an ICS was deemed essential. Even in rural households, cooking in a separate kitchen and transporting food to the main house was considered difficult specially during rainy season and when members felt exhausted and this also motivated them to use ICS within the main house [55]. Having a space for kitchen inside the main house was likely to increase the adoption of ICS and cleaner fuels [97]. The use of ICS could also be affected by the indoor characteristics of the kitchen such as those with indoor separate kitchen, indoor with attached kitchen, indoor with no separate kitchen area. Traditional stoves have an additional space requirement and require the engagement of several family members, especially in rural households. The prolonged cooking time required for traditional stoves offered women and family members socialize around the cooking area, allocating a time and space for engagement not so different from the modern ‘kitchen island’ increasingly essential for suburban homes in high income countries (HICs) [41, 43, 72, 77, 99]. Lack of a clean energy supply chain was found to make a major impact on adoption of ICS [75]. Some studies interpreted the use of LPG as a sign of women empowerment as it meant reducing the time required for the maintenance of traditional stoves [57].

**Cost of fuel

10. On average, using clean energy such as LPG meant households reported a 30% reduction in fuel expenses [24].

Comment: Are the authors sure that the cost of LPG would be same across different countries? This particular reference may show 30%. Due to the varying cost the reduction in fuel expenses may vary. Emission reduction percentage could be accepted as this is not going to be different irrespective of the location of combustion. Still it may vary due to the variations in the composition of LPG in different countries. Please clarify with some changes.**

**AUTHORS:** Thank you for the suggestion. We have revised the statement to clarify the limitation of average reduction in cost saving from adoption of LPG.

**Supply-chain feasibility
11. In India, 45% of rural households were found to face difficulties in obtaining regular LPG cylinder refills [24].

Comment: In India through the Pradhan Mantri Ujjwala Yojana (PMUY) national scheme, nearly 100 million free LPG stoves and new connections are provided. Moreover, the LPG distributor network is well established for more than several decades. Hence, there should no difficulties in obtaining cylinder refills. There should be other reason such as illegal market or underground economy. Recent references will help better understand the situation. The author shall clarify this.**

**AUTHORS:** Thank you for the suggestion. We agree with your suggestion, we have revised and added the latest data around Pradhan Mantri Ujjwala Yojana and how it has enhanced the access. We have revised under the heading ‘Supply-chain feasibility’ sub-heading ‘supplies’ and reads as follows:

The limited availability of LPG required travel to refill the cylinders, direct and indirect costs associated with travel, and safety concerns were likely to discourage the adoption of ICS [22-26, 40, 42, 45, 47, 48, 50, 53, 56, 63, 71, 87, 94, 97, 98, 101, 103, 106]. Easy availability of cookstove (AOR 1.81; 95% CI 1.5–12.17) made adoption more likely [34, 39]. In India, The Pradhan Mantri Ujjwala Yojana (PMUY) distributed over 80 million new LPG stoves across the country between 2016 and 2019 and well over 100 million new LPG stoves in 2023 [110]. Under this continuing national subsidy-scheme, consumers pay for the stove and the cylinder deposit and reduced the upfront cost. This has increased the access of LPG to over 95% of households [70]. In Nepal, availability was found to be compromised by limited energy infrastructure, including poor and intermittent access to LPG refills, and affected the adoption and use [76]. A study from Uganda highlighted the feasibility and benefit of building the ICS locally through the provision of training to community members, and use of locally available resources which could resolve the supply and dependence issues [43]. Lack of coordination, poor regulation, and insufficient market development were found to affect the supply of ICS negatively [34].

Also, more at 7^th^ paragraph in Discussion section that reads as follows:

Government policies regulating supplies and promotion of the energy sources can have significant impact on the uptake of ICS [123, 124]. Availability, accessibility, and affordability are fundamental contributors in adopting a new technology, and thus any impediments to these critical elements affect the adoption and sustained use of ICS [114, 115]. Policy engagement on the need of clean energy such as subsidies had a major impact on the adoption and sustained use of LPG stoves [70, 75, 94, 110]. Government’s initiatives on cleaner energy, had a major impact on adoption of ICS in rural China [75]. India’s The Pradhan Mantri Ujjwala Yojana (PMUY) increased the access to LPGs to more than 100 million households [70, 110] and Indonesia’s ‘Zero-kero Programme’ achieved a fivefold increase in LPG consumption [94]. Achieving nationwide scale-up and sustainable adoption of LPG relies on sustained governmental initiatives to ensure accessibility, financial support, and active engagement with the population [125]. Despite government’s aim to promote cleaner energy (LPG and electricity), 80% of population in rural Nepal still use solid-biomass fuels, partly because of the absence of consistent access to LPGs and subsidy scheme by the government [126, 127]. The rural versus urban disparity in use of LPG was apparent in many countries [23, 55, 57, 87, 105]. For instance, in Peru, the main barrier to adopting LPG was the availability and access to the LPG in rural villages [103]; and thus requires a focused and tailored approach in ensuring the access, and subsidies. A recent review echoes how government’s policy initiatives are critical to ensure the access, affordability and adoption of LPGs [116].

**Policy
12. While the policies in few countries are reviewed, the Pradhan Mantri Ujjwala Yojana (PMUY) national scheme for providing free LPG stove and connections in India is not reviewed. Please clarify.**

**AUTHORS:** Thank you for the suggestion. We have revised the section and have added about the PMUY and its impact in adoption of LPG in India.

India’s The Pradhan Mantri Ujjwala Yojana (PMUY) offers another example on how a government subsidy scheme enhanced the use of cleaner fuel (LPG) increasing the nationwide access [70, 110].

**General comments:
13. This scoping review largely have focused on LPH as ICS, other types of Biomass ICS have not been reviewed. ICS includes all categories of stoves and fuel. This review looks biased toward one type of stove. The author shall clarify this.**

**AUTHORS:** We agree with your analysis. This is partly due to the nature of studies reporting on ICS where majority pointed towards the LPG stove as a more preferred form of ICS, although ‘ICS’ can range in a spectrum which we have outlined in the introduction section. We have also acknowledged this as a limitation within discussion section under the heading ‘Strengths and limitations’.

The term ‘ICS’ used in this review specifically meant switching of traditional stove to any advanced categories, this may have generalized the information related to specific stove categories. In addition, because bulk of the literature reported the LPG stove as the ICS, our analysis on acceptability was focused on LPG stove and may have generalized the findings. Although this review explored factors affecting acceptability of ICS operating at various levels, future research could explore more on how specific types of ICS have varying impacts on adoption and sustained use over time.

**14. The author rightly mentioned the barrier to use LPG stove was availability and the cost in the “Cost of fuel” section.

Comment: In this or in the staking section, I have not observed any literatures discussing the purpose or preferences of using LPG. Generally due to the high cost, people use LPG to boil water, milk or heating that required immediate use rather than using biomass stove. This has not been discussed in this review. Please clarify.**

**AUTHORS:** Thank you for the suggestion. We have revised the ‘cost of fuel’ section to clarify how LPG stoves were used in certain conditions such as when preparing food during busy hours, including boiling milk, water or tea for immediate purposes. We have also added the reference in ‘stacking section’ to reinforce the statement.

Revised statement at ‘Cost of fuel’ section that reads as follows:

The main barrier to the use of the LPG stove was the availability and cost of LPG refills. For instance, the cost was found to restrain its widespread use, reserving its use for cooking food during busy hours, preparing tea, boiling water or milk for immediate use [82].

**15. The author has not reviewed any emission standard for the ICS. The author shall narrate on this. Please clarify.**

**AUTHORS:** Thank you for the suggestion. Because we undertook this review to explore the acceptability of ICS; the emission standard may have been translated among the factors presented such as how emissions by more traditional stoves may have affected their health.

**16. Reduction in pollution concentration or exposures could also be a factor to switch over to ICS. Many ICS studies are published. The authors shall include a section on this as this will be within the scope of research question. Please clarify.**

**AUTHORS:** Thank you for the suggestion. We agree that air pollution concentration could be a factor to switch over to ICS. Instead of exploring the acceptability based on the association between pollution concentration and the choice of ICS, we explored the factors affecting acceptability as reported in terms of ‘health benefits’. Secondly, residents or the stove users may translate the role of pollution (or indoor/household air pollution) in terms of how it affects their health than being well versed in scientific concept of how traditional stoves contribute to HAP. These are also reflected in section e.g. Policy, where the last statement of the first paragraph emphasizes the need to have widespread initiative in highlighting the role of the household in indoor air pollution related ill health and reads as follows:

Government-led information, communication, and education initiatives to highlight the role of the household in indoor air pollution related ill-health was effective in promoting the use of ICS in Nepal [76].

Indeed, lack of awareness on household air pollution related to their impact on health were often reported. For instance a statement within the section ‘Health benefits’ reads as follows:

In contrast, lack of awareness on what household air pollution is and how it affected health were found to be a major barrier to adopting cleaner fuels [37, 41, 66, 67]. The low back pain when collecting firewood for traditional stove triggered some respondents to switch to ICS [101]. In contrast, the perception that smoke can cure food [smoke was perceived to maintain hygiene], deter insects and other pests tended to support the continued use of traditional stoves [77].

**Reviewer #2**

**Thank you for the submission. While the topic is important and timely, I have some major concerns:
1. While the manuscript mentions an overarching goal to explore the factors affecting ICS acceptability, it does not clarify how these factors will be systematically examined or which specific outcomes (e.g., health, economic, or social impacts) are being prioritized. Additionally, it does not differentiate between initial adoption and sustained use.**

**AUTHORS:** Thank you for the suggestion. Although these broad drivers at different layers seem to be illustrated as descriptively as possible to present the multi-level and dynamics within the context of ICS acceptability, we have focused on how policy subsidy and broadly, cost-reduction measures can become successful, for instance, the examples from Indonesia and India. Much of the literature distils down to the affordability of the ICS and fuel types, and thus any measures taken towards mitigating this main barrier seem to affect the coverage and adoption (with stacking in combination) of ICS. Since our research question attempted to explore all the drivers of acceptability of ICS and at various levels, we wanted to retain the landscape of drivers as it emerged from the literature.

Related to adoption and sustained use, you rightly point out the differentiation between initial adoption and sustained use. This was partly due to the nature of literature which did not adequately assess the longitudinal adoption (sustained use) of ICS. Moreover, much of the literature described how stacking was common that obscured the sustained use of particular ICS, instead presented on the dual/multiple use of energy resources or ICS. We have also reflected these limitations in revised ‘strengths and limitations’ within ‘Discussion’ section.

**2. The manuscript relies heavily on qualitative themes, though quantitative evaluations exist, especially on health outcomes and cost savings. Integrating both qualitative and quantitative evidence would strengthen the findings.**

**AUTHORS:** Thank you for your evaluation. We agree that the manuscript relies on qualitative themes. As you are aware that ‘acceptability’ is a social science concept that explores attitudinal factors towards the particular intervention or technology, majority of the articles retained for analysis had qualitative components, even when they adopted mixed methods. We could have more quantitative articles if we had an additional research question or more specific research question, such as exploring relationship between health effects and acceptability, or cost of fuel and acceptability. Nonetheless, in our review, we have had few quantitative articles (supplementary S3 file) and have included them into the manuscript. We have also reflected this as a limitation in ‘strengths and limitations’ section and reads as follows:

While initial adoption and sustained use of ICS are often not delineated in the literature, in part because these issues often co-exist as explained by the stacking of multiple energy sources, disentangling factors affecting stacking versus use of specific ICS and type of fuel could highlight the drivers of particular ICS and fuel types. In future, policy analysis could further highlight the impact on acceptability of ICS.

**3. Adoption factors are described but not sufficiently grounded in theoretical frameworks. While the “energy ladder” and “energy stacking” theories are mentioned, these could be explored in more depth. Incorporating frameworks like the Technology Acceptance Model and COM-B could enhance coherence in discussing adoption behaviors.**

**AUTHORS:** Thank you for the suggestion. First of all, energy ladder and energy stacking were found to be the most relevant and reported concepts in explaining the adoption of ICS. To our knowledge, these two main concepts offer pertinent response to the question of acceptability, thus we retained them in the background section. We agree with you that there are multifarious social models and theories explaining the adoption of innovation or technology, nonetheless, as you are aware all of these models have limitations in fully explaining the adoption (and sustained use) [1, 2]. Majorities are more inclined towards the individual level behavioural change, while others such as innovation of diffusion is more inclined towards wider institutional (and policy context) on how new technologies could be widely implemented at population level [1]. TAM does have some overlap with the innovation of diffusion, but again focuses on the individual level behavioural element that is perceived usefulness and perceived ease of use, which omits the remit of social, and structural factors in shaping the adoption at population level [3, 4]. We chose to derive explanation using diffusion model, because we were interested in explaining how the adoption of technology (ICS) can distribute over various categories (early adopters to late adopters) and how wider social factors (e.g. policy) affect it. We have revised the discussion section to incorporate your concern.

COM-B model could be a useful explanatory framework when focusing on individual level (behavioural) factors affecting the adoption of a technology or ICS, but again does not fully explain the wider structural factors. A recent formative study conducted in Guatemala showed how COM-B model was able to explain the pregnant women’s behaviour in adopting the ICS, but largely overlooks the wider ecological factors, e.g. access and availability [5].

The review of theories related to ICS also resonated how structural determinants were omitted when using these theoretical frameworks [2]. Relevant to our acceptability as shown in figure 2 was to illustrate the ecological perspective (social level factors) how multi-layered factors explain such a complex social process of decision making.

Based on your suggestion, we have revised the second paragraph within the Discussion section on this and reads as follows:

Two broad concepts were described a) the energy ladder that proposes use of energy to be dependent on economic caliber and b) stacking fuel for security purposes [11]. In practice both approaches are operating in concerted fashion ultimately promoting adoption of multiple sources of fuels (**Fig 4**). Adopting an ICS is a complex social process and operate at multiple levels from individual to institutional and policy within the context [111, 112]. Nonetheless, several theories aiming at individual behavioural interventions are limited by their scope as acceptability and adoption of ICS are inevitably shaped by broader context of social, and policy structures [111]. In a wider social and political context, acceptability of an improved cook stove is thus a complex social process echoing the elements of ‘theory of adoption’ and ‘innovation diffusion process’. Both entail development and commercialization of an innovation, followed by the diffusion and adoption of the innovation by the users and its consequences including the critical roles of policy and social support [5, 113]. Five stages of the innovation decision process involve steps of disseminating knowledge and persuasion to decision making followed by implementation and confirmation (practiced through reflection on whether or not to sustain the use) [12, 113]. The decision-making steps when using novel equipment or technology are also echoed across the discipline. For instance, adoption of a new product in medical sciences and health care are regulated by WHO’s policy on biomedical products that are assessed based on their availability, accessibility, appropriateness, and affordability [114, 115].

**4. While the manuscript includes studies from Africa, Asia, and South America, it lacks regional comparisons. Comparing regional adoption rates, barriers, and enablers would improve understanding of how context shapes ICS acceptability.**

**AUTHORS:** Thank you for your comment. While true that manuscript does include studies from Africa, Asia and South America, we synthesized the findings in terms of commonalities of factors/themes, their prominence and the implications rather than making a comparative analysis. Aware of our research question and scope of the current work, we attempted to draw on drivers of acceptability of ICS and restrict the comparative analysis because of the differences in social, political and geographical context. Nonetheless, to illustrate the policy landscape which can have major impact on the acceptability of the ICS (based on our findings), we have revised the paragraph in Discussion section focusing on the impacts related to ICS access and adoption in Asia. Additional information categorized explicitly as barriers and enablers (are presented in supplementary file 3) of ICS informed the content of this review.

The 7th paragraph in Discussion section is revised as follows:

Government policies regulating supplies and promotion of the energy sources can have significant impact on the uptake of ICS [123, 124]. Availability, accessibility, and affordability are fundamental contributors in adopting a new technology, and thus any impediments to these critical elements affect the adoption and sustained use of ICS [114, 115]. Policy engagement on the need of clean energy such as subsidies had a major impact on the adoption and sustained use of LPG stoves [70, 75, 94, 110]. Government’s initiatives on cleaner energy, had a major impact on adoption of ICS in rural China [75]. India’s The Pradhan Mantri Ujjwala Yojana (PMUY) increased the access to LPGs to more than 100 million households [70, 110] and Indonesia’s ‘Zero-kero Programme’ achieved a fivefold increase in LPG consumption [94]. Achieving nationwide scale-up and sustainable adoption of LPG relies on sustained governmental initiatives to ensure accessibility, financial support, and active engagement with the population [125]. Despite government’s aim to promote cleaner energy (LPG and electricity), 80% of population in rural Nepal still use solid-biomass fuels, partly because of the absence of consistent access to LPGs and subsidy scheme by the government [126, 127]. The rural versus urban disparity in use of LPG was apparent in many countries [23, 55, 57, 87, 105]. For instance, in Peru, the main barrier to adopting LPG was the availability and access to the LPG in rural villages [103]; and thus requires a focused and tailored approach in ensuring the access, and subsidies. A recent review echoes how government’s policy initiatives are critical to ensure the access, affordability and adoption of LPGs [116].

**5. The methodology lacks transparency on study inclusion/exclusion.**

**AUTHORS:** Thank you for the suggestion. We have added the inclusion/exclusion criteria within the methodology section under the heading ‘Collating of data, synthesizing, and reporting of the findings’ and reads as follows:

Articles were excluded if 1. they were in languages other than English; 2. briefly mentioned ICS without adequate content on acceptability; 3. reported as comparative accounts of clean energy sources without adequate details on improved cooking stoves and 4. improved cooking stoves were assessed for their functionalities (e.g. cooking efficiency, and emission) without users’ preference. All articles regardless of their types with relevant and adequate content addressing our research questions were included in a full text analysis.

**6. The policy recommendations are too broad. More context-specific recommendations with evidence on the most effective policies or subsidies would make the conclusions more actionable.**

**AUTHORS:** Thank you for the suggestion. Based on what we found in this review, we have revised the discussion section. We have distilled out the policies that have made major impacts in the adoption of LPG from Indonesia, India and China in the revised ‘Discussion’ section and reads as follows:

Government policies regulating supplies and promotion of the energy sources can have significant impact on the uptake of ICS [123, 124]. Availability, accessibility, and affordability are fundamental contributors in adopting a new technology, and thus any impediments to these critical elements affect the adoption and sustained use of ICS [114, 115]. Policy engagement on the need of clean energy such as subsidies had a major impact on the adoption and sustained use of LPG stoves [70, 75, 94, 110]. Government’s initiatives on cleaner energy, had a major impact on adoption of ICS in rural China [75]. India’s The Pradhan Mantri Ujjwala Yojana (PMUY) increased the access to LPGs to more than 100 million households [70, 110] and Indonesia’s ‘Zero-kero Programme’ achieved a fivefold increase in LPG consumption [94]. Achieving nationwide scale-up and sustainable adoption of LPG relies on sustained governmental initiatives to ensure accessibility, financial support, and active engagement with the population [125]. Despite government’s aim to promote cleaner energy (LPG and electricity), 80% of population in rural Nepal still use solid-biomass fuels, partly because of the absence of consistent access to LPGs and subsidy scheme by the government [126, 127]. The rural versus urban disparity in use of LPG was apparent in many countries [23, 55, 57, 87, 105]. For instance, in Peru, the main barrier to adopting LPG was the availability and access to the LPG in rural villages [103]; and thus requires a focused and tailored approach in ensuring the access, and subsidies. A recent review echoes how government’s policy initiatives are critical to ensure the access, affordability and adoption of LPGs [116].

**7. The manuscript does not adequately address sustainability challenges, such as scaling up LPG.**

**AUTHORS:** Thank you for the suggestion, again this may be because of our research question, that broadly looked at the acceptability of ICS and may have limited the literature evaluating the sustainability challenges including scaling up of LPGs. Nonetheless, we have revised the discussion section to incorporate how scaling up of LPGs require a broad nationwide initiative within the context of successful policies that have increased the access and sustained use of LPGs. We have also reflected this as a limitation of our review within the discussion section and reads as follows:

Although this review explored factors affecting acceptability of ICS operating at various levels, future research could explore more on how specific types of ICS have varying impacts on adoption and sustained use over time. While initial adoption and sustained use of ICS are often not delineated in the literature, in part because these issues often co-exist as explained by the stacking of multiple energy sources, disentangling factors affecting stacking versus use of specific ICS and type of fuel could highlight the drivers of particular ICS and fuel types. In future, policy analysis could further highlight the impact on acceptability of ICS.

**8. Gender and equity issues are not adequately addressed. How gender dynamics, income inequality, and financial access affect ICS adoption could be discussed in more depth.**

**AUTHORS:** Thank you for the suggestion. We did find gender dynamics, income level and financial access affecting the ICS adoption as presented in results section. We attempted to discuss the broader implications of our findings and thus distil down the findings in Discussion section preventing the repetition. Based on your suggestion, we have added the role of gender dynamics, income and financial access affecting the acceptability and adoption of the ICS. The fourth paragraph in Discussion section reads as follows:

End-users were the ultimate decision makers of ICS, but their socio-demographic characteristics such as their educational level, economic caliber, and decision-making power at household level including the role of gender affected the adoption of an ICS [9, 12, 34, 41, 81]. Although women are often responsible for household tasks, including kitchen duties and cooking, their role in deciding to use ICS was limited, with the decision frequently made by the male income earner, particularly in the context of cultural patriarchy, and access to financial resources [67, 77, 83]. This suggests the need to design interventions incorporating gender and equity issues in LMICs to give women agency in choosing their preferred stove [83, 116]. An individual was likely to choose the type of stove based on the time consumed by the given stove, the quantity of food required, the specific dish (e.g. traditional dishes), social aspects (kitchen as a hub for socialization in a house) and finally the cultural demands of a typical kitchen (taste of food, and alignment with tradition) [41, 43, 72, 77, 99]. The health benefits of ICS and cleaner energy were increasingly recognized and the transition to cleaner energy seemed to be driven largely by the health benefits (or health hazards by the firewood) [27].

**9. More contextualization is needed, e.g., are the barriers to adoption different in urban vs. rural settings?**

**AUTHORS:** Thank you for the suggestion. We agree that there are rural-urban divides in the adoption of ICS. There were number of studies highlighting the issue and we included them extensively in the result section. We have revised the discussion section to briefly highlight the issue on need of contextualized approaches to redress the rural-urban divide in use of LPG. Rural regions bear the brunt of barriers, for instance, geographical terrain, means of transport, economy—all can affect the availability, access and sustainability and this distils down to access issues in most countries, thus we have highlighted this with few examples from Nepal, and Peru and reads as follows:

Achieving nationwide scale-up and sustainable adoption of LPG relies on sustained governmental initiatives to ensure accessibility, financial support, and active engagement with the population [125]. Despite government’s aim to promote cleaner energy (LPG and electricity), 80% of population in rural Nepal still use solid-biomass fuels, partly because of the absence of consistent access to LPGs and subsidy scheme by the government [126, 127]. The rural versus urban disparity in use of LPG was apparent in many countries [23, 55, 57, 87, 105]. For instance, in Peru, the main barrier to adopting LPG was the availability and access to the LPG in rural villages [103]; and thus requires a focused and tailored approach in ensuring the access, and subsidies. A recent review echoes how government’s policy initiatives are critical to ensure the access, affordability and adoption of LPGs [116].

**10. The manuscript could better emphasize research gaps and suggest future research directions.**

**AUTHORS:** Thank you for the suggestion. We have added the research gaps and future research directions highlighted in our ‘strengths and limitations’ section and reads as follows:

The term ‘ICS’ used in this review specifically meant switching of traditional stove to any advanced categories, this may have generalized the information related to specific stove categories. In addition, because bulk of the literature reported the LPG stove as the ICS, our analysis on acceptability was focused on LPG stove and may have generalized the findings. Although this review explored factors affecting acceptability of ICS operating at various levels, future research could explore more on how specific types of ICS have varying impacts on adoption and sustained use over time. While initial adoption and sustained use of ICS are often not delineated in the literature, in part because these issues often co-exist as explained by the stacking of multiple energy sources, disentangling factors affecting stacking versus use of specific ICS and type of fuel could highlight the drivers of particular ICS and fuel types. In future, policy analysis could further highlight the impact on acceptability of ICS.

6. PLOS authors have the option to publish the peer review history of their article ([what does this mean?](https://url.uk.m.mimecastprotect.com/s/C1yTCRMYWCv0OmXcOsjF1MUHo?domain=track.editorialmanager.com)). If published, this will include your full peer review and any attached files.


**Do you want your identity to be public for this peer review?** If you choose “no”, your identity will remain anonymous but your review may still be made public.


For information about this choice, including consent withdrawal, please see our [Privacy Policy](https://url.uk.m.mimecastprotect.com/s/JlMaCV5p1cxkZw8uQtZFExBHU?domain=track.editorialmanager.com).

Reviewer #1: **Yes:** Sankar Sambandam

Reviewer #2: **Yes:** Rachit Sharma

[NOTE: If reviewer comments were submitted as an attachment file, they will be attached to this email and accessible via the submission site. Please log into your account, locate the manuscript record, and check for the action link "View Attachments". If this link does not appear, there are no attachment files.]

While revising your submission, please upload your figure files to the Preflight Analysis and Conversion Engine (PACE) digital diagnostic tool, [https://pacev2.apexcovantage.com/](https://url.uk.m.mimecastprotect.com/s/IebSCWwO2C5X498cpu1FoPcXY?domain=pacev2.apexcovantage.com). PACE helps ensure that figures meet PLOS requirements. To use PACE, you must first register as a user. Registration is free. Then, login and navigate to the UPLOAD tab, where you will find detailed instructions on how to use the tool. If you encounter any issues or have any questions when using PACE, please email PLOS at [figures@plos.org](mailto:figures@plos.org). Please note that Supporting Information files do not need this step.

**References**

1.	Taherdoost H: **A review of technology acceptance and adoption models and theories.** *Procedia manufacturing* 2018, **22:**960-967.

2.	Del Rio DDF, Lambe F, Roe J, Matin N, Makuch KE, Osborne M: **Do we need better behaved cooks? Reviewing behavioural change strategies for improving the sustainability and effectiveness of cookstove programs.** *Energy Research & Social Science* 2020, **70:**101788.

3.	Holden RJ, Karsh B-T: **The technology acceptance model: its past and its future in health care.** *Journal of biomedical informatics* 2010, **43:**159-172.

4.	Marangunić N, Granić A: **Technology acceptance model: a literature review from 1986 to 2013.** *Universal access in the information society* 2015, **14:**81-95.

5.	Thompson LM, Diaz-Artiga A, Weinstein JR, Handley MA: **Designing a behavioral intervention using the COM-B model and the theoretical domains framework to promote gas stove use in rural Guatemala: a formative research study.** *BMC public health* 2018, **18:**1-17.
